# Eurythmy therapy in chronic disease: a four-year prospective cohort study

**DOI:** 10.1186/1471-2458-7-61

**Published:** 2007-04-23

**Authors:** Harald J Hamre, Claudia M Witt, Anja Glockmann, Renatus Ziegler, Stefan N Willich, Helmut Kiene

**Affiliations:** 1 Institute for Applied Epistemology and Medical Methodology, Böcklerstr. 5, 79110 Freiburg, Germany; 2 Institute of Social Medicine, Epidemiology, and Health Economics, Charité University Medical Center, Campus Mitte, 10098 Berlin, Germany; 3 Society for Cancer Research, Kirschweg 9, 4144 Arlesheim, Switzerland

## Abstract

**Background:**

Many patients with chronic diseases use complementary therapies, often provided by their physicians. In Germany, several physician-provided complementary therapies have been reimbursed by health insurance companies as part of health benefit programs. In most of these therapies, the patient has a predominantly passive role. In eurythmy therapy, however, patients actively exercise specific movements with the hands, the feet or the whole body. The purpose of this study was to describe clinical outcomes in patients practising eurythmy therapy exercises for chronic diseases.

**Methods:**

In conjunction with a health benefit program, 419 outpatients from 94 medical practices in Germany, referred to 118 eurythmy therapists, participated in a prospective cohort study. Main outcomes were disease severity (Disease and Symptom Scores, physicians' and patients' assessment on numerical rating scales 0–10) and quality of life (adults: SF-36, children aged 8–16: KINDL, children 1–7: KITA). Disease Score was documented after 0, 6 and 12 months, other outcomes after 0, 3, 6, 12, 18, 24, and (SF-36 and Symptom Score) 48 months.

**Results:**

Most common indications were mental disorders (31.7% of patients; primarily depression, fatigue, and childhood emotional disorder) and musculoskeletal diseases (23.4%). Median disease duration at baseline was 3.0 years (interquartile range 1.0–8.5). Median number of eurythmy therapy sessions was 12 (interquartile range 10–19), median therapy duration was 119 days (84–188).

All outcomes improved significantly between baseline and all subsequent follow-ups (exceptions: KITA Psychosoma in first three months and KINDL). Improvements from baseline to 12 months were: Disease Score from mean (standard deviation) 6.65 (1.81) to 3.19 (2.27) (p < 0.001), Symptom Score from 5.95 (1.75) to 3.49 (2.12) (p < 0.001), SF-36 Physical Component Summary from 43.13 (10.25) to 47.10 (9.78) (p < 0.001), SF-36 Mental Component Summary from 38.31 (11.67) to 45.01 (11.76) (p < 0.001), KITA Psychosoma from 69.53 (15.45) to 77.21 (13.60) (p = 0.001), and KITA Daily Life from 59.23 (21.78) to 68.14 (18.52) (p = 0.001). All these improvements were maintained until the last follow-up. Improvements were similar in patients not using diagnosis-related adjunctive therapies within the first six study months.

Adverse reactions to eurythmy therapy occurred in 3.1% (13/419) of patients. No patient stopped eurythmy therapy due to adverse reactions.

**Conclusion:**

Patients practising eurythmy therapy exercises had long-term improvement of chronic disease symptoms and quality of life. Although the pre-post design of the present study does not allow for conclusions about comparative effectiveness, study findings suggest that eurythmy therapy can be useful for patients motivated for this therapy.

## Background

In the developed world the most frequent reason for people to seek health care is a chronic disease [1]. Chronic diseases are the most common cause of disease burden worldwide, are often associated with comorbidity, and are rarely completely cured [1]. Strategies to improve the outcome of chronic diseases include drug regimens, enhanced healthcare provision, and patient self-management programs [2-4]. Many patients with chronic disease also use complementary therapies [5,6], often provided by their physicians. In Germany, several physician-provided complementary therapies have been reimbursed by health insurance companies as part of special health benefit programs ("Modellvorhaben") [7-10]. In most of these complementary therapies the physician is the active person, directly treating (e.g. giving acupuncture) or prescribing therapy (e.g. homoeopathic medications), while the patient has a predominantly passive role. Anthroposophic medicine (AM, a complementary system of medicine founded by Rudolf Steiner and Ita Wegman [11]), includes two interventions that require the patient to engage in active exercises: AM art and eurythmy therapy.

Eurythmy therapy (EYT, Greek: eurythmy = "harmonious rhythm") is an exercise therapy involving cognitive, emotional, and volitional elements [12]. EYT is prescribed by AM physicians and provided by EYT therapists in individual or small group sessions during which patients are instructed to perform specific movements with the hands, the feet or the whole body. EYT movements are related to the sounds of vowels and consonants, to music intervals or to soul gestures, e.g. sympathy-antipathy. For each patient one or several movements are selected, depending on the patient's disease, his constitution, and on the EYT therapist's observation of the patient's movement pattern. This selection is based on a core set of principles, prescribing specific EYT movements for specific diseases, constitutional types, and movement patterns [13,14].

EYT sessions usually last 45 min; between therapy sessions patients practice EYT exercises daily [14]. An EYT therapy cycle usually consists of 12–15 sessions. EYT can be used as monotherapy or combined with other AM therapies. Qualification as an EYT therapist requires 5 1/2 years of training according to an international, standardised curriculum. EYT is presently provided by approximately 1,550 therapists in 31 countries worldwide (A. Jaschke, International Coordination AM, personal communication, February 2007). Half of EYT therapists work in Germany or Switzerland. In these two countries EYT costs ca. 40 Euro per session and is covered by many health insurance companies. In other countries costs vary and are not covered by health insurance.

EYT is believed to have both general effects (e.g. improving breathing patterns and posture, strengthening muscle tone, enhancing physical vitality [15]) and disease-specific effects [14]. Observational studies suggest that EYT and other AM therapies can be useful for a variety of clinical conditions [12,16-25]. However, all these studies were monocentric, all but one [12] evaluated multimodal AM therapy including EYT in only a proportion of the patients, and all but three studies [18-20] had a sample size of less than 25 AM patients. Here we present a multi-centre long-term study of EYT with 419 patients.

## Methods

### Study design and objective

This is a prospective four-year cohort study in a real-world medical setting. The study was part of a research project on the effectiveness and costs of AM therapies in outpatients with chronic disease (Anthroposophic Medicine Outcomes Study, AMOS) [8,26]. The AMOS project was initiated by a health insurance company in conjunction with a health benefit program and included the following effectiveness issues:

1) Are AM therapies in general associated with clinically relevant improvements of chronic diseases? (see [8])

2) Are specific AM therapies (such as EYT) associated with such improvements?

3) If yes: To which extent are these improvements found in different age, gender, and diagnostic subgroups?

4) How do improvements of specific diagnostic groups compare to improvements with other interventions?

The issues 2 and 3 were addressed in this EYT analysis, the objective of which was to study symptoms, quality of life, adjunctive therapies, health service use, adverse reactions, and therapy satisfaction in outpatients with chronic diseases receiving EYT under routine clinical conditions. EYT was evaluated as a therapy package, including physician- and therapist-patient interactions.

### Setting, participants and therapy

All physicians certified by the Physicians' Association for Anthroposophical Medicine in Germany and working in an office-based practice or outpatient clinic in Germany were invited to participate in the study. The participating physicians were instructed to enrol consecutive patients fulfilling eligibility criteria. Inclusion criteria were (1) outpatients aged 1–75 years, (2) referral to EYT for any indication (main diagnosis). Exclusion criteria were (1) previous EYT for main diagnosis, (2) ongoing EYT.

Participating EYT therapists were certified by the Eurythmy Therapy Association of Germany. EYT was administered at the discretion of the physicians and EYT therapists.

### Clinical outcomes

• Disease severity was assessed on numerical rating scales [27] from 0 („not present“) to 10 („worst possible“): Disease Score (physician's global assessment of severity of main diagnosis, documented in patients enrolled up to 30 Sep 2000); Symptom Score (patients' assessment of one to six most relevant symptoms present at baseline, documented in patients enrolled after 1 Jan 1999).

• Quality of life was assessed with SF-36^® ^Physical and Mental Component Summary Measures, the eight SF-36 subscales, and the SF-36 Health Change item [28] for adults; with KINDL^® ^40-item version, Summary Score and four subscales [29] for children 8–16 years; and with KITA Psychosoma and Daily Life subscales [30] for children 1–7 years.

Disease Score was documented after 0, 6 and 12 months, other clinical outcomes after 0, 3, 6, 12, 18, 24, and (Symptom Score and SF-36) 48 months.

### Other outcomes

• Adjunctive therapy and health service use in the pre-study year was documented at study enrolment, use in the first study year was documented after six and 12 months, and use in the second study year was documented after 18 and 24 months. Items were: medication (additional documentation after three months), physician and dentist visits, paraclinical investigations, inpatient hospital and rehabilitation treatment, surgeries, physiotherapy, ergotherapy, psychotherapy, Heilpraktiker (non-medical practitioner) visits, and sick leave.

• Use of diagnosis-related adjunctive therapies within the first six study months was analysed in patients with a main diagnosis of mental, respiratory or musculoskeletal diseases, or headache disorders. Diagnosis-related therapies were any of the following therapies, if used for at least one day per month: Mental diseases: psychotherapy (in children ergotherapy or play therapy), antiepileptic, psycholeptic, analeptic, and anti-addiction drugs (ATC-Index N03A, N05–06, N07B); Respiratory diseases: relevant drugs (H02, J01–02, J04–05, J07A, L03, R01, R03, R06–07) or surgery; Musculoskeletal diseases: immunosuppressive, musculoskeletal, analgesic and antidepressant drugs (L04, M01–05, M09, N02A-B, N06A), physiotherapy or relevant surgery; Headache disorders: analgesics, antimigraine drugs and antidepressants (C04AX01, C07AA05, C07AB02, C08CA06, C08DA01, N02, N03AG01, N06A, N07CA03).

• Therapy ratings were documented after six and 12 months: Patient rating of therapy outcome, patient satisfaction with therapy, EYT effectiveness rating by patient and physician.

• Adverse drug or therapy reactions were documented during the first 24 study months: cause, intensity (mild/moderate/severe = no/some/complete impairment of normal daily activities); Serious Adverse Events (physician documentation).

### Data collection

All data were documented with questionnaires sent in sealed envelopes to the study office. Physicians documented eligibility criteria; therapists documented EYT administration; all other items were documented by patients (by caregivers of children < 17 years) unless otherwise stated. Patient responses were not made available to physicians. Physicians were compensated €40 per included and fully documented patient, while patients received no compensation.

Data were entered twice by two different persons into Microsoft^® ^Access 97. The two datasets were compared and discrepancies resolved by checking with the original data.

### Quality assurance, adherence to regulations

The study was approved by the Ethics Committee of the Faculty of Medicine Charité, Humboldt University Berlin, and was conducted according to the Helsinki Declaration and the International Conference on Harmonisation Good Clinical Practice guidelines. Written informed consent was obtained from all patients before enrolment.

### Data analysis

Data analysis (SPSS^® ^13.0.1, StatXact^® ^5.0.3) was performed on all patients fulfilling eligibility criteria. For continuous data the Wilcoxon Signed-Rank test was used for paired samples and the Mann-Whitney U-test for independent samples; median differences with 95% confidence intervals (95%-CI) were estimated according to Hodges and Lehmann [31]. For binominal data McNemar test and Fisher's exact test were used. All tests were two-tailed. Significance criteria were p < 0.05 and 95%-CI not including 0. Pre-post effect sizes were calculated as Standardised Response Mean (= mean change score divided by the standard deviation of the change score) and were classified as small (0.20–0.49), medium (0.50–0.79), and large (≥ 0.80) [32]. Unless otherwise stated, therapies and health services were analysed in patients enrolled after 1 Jan 1999 with at least three out of five follow-ups available; for each item and follow-up period, missing values were replaced by the group mean value. Clinical outcomes were analysed in patients with evaluable data for each follow-up, without replacement of missing values.

## Results

### Participating physicians and therapists

101 physicians screened patients referred to EYT. 94 physicians enrolled patients into the study; these physicians did not differ significantly from all AM-certified physicians in Germany (n = 362) regarding gender, age, number of years in practice, and the proportion of primary care physicians. Patients were treated by 118 EYT therapists. Comparing these therapists to certified EYT therapists without study patients (n = 231), no significant differences were found regarding gender or age. Median number of years since EYT school graduation was 9.0 years for therapists with study patients and 13.0 years for therapists without study patients (median difference 2.0 years; 95%-CI 1.0–4.0 years; p = 0.005).

### Patient recruitment and follow-up

**Figure 1:**
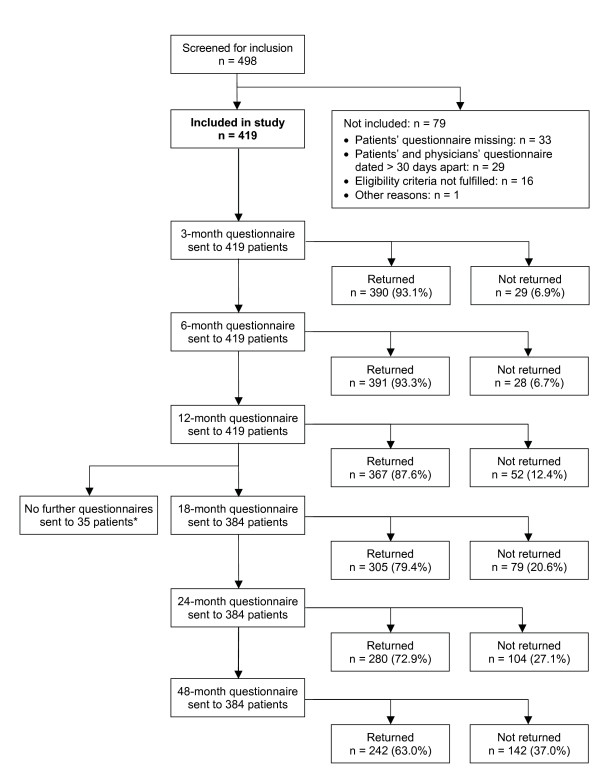
**Patient recruitment and follow-up**. *18-, 24-, and 48-month follow-up questionnaires were not sent to patients enrolled before 1 Jan 1999.

From 1 July 1998 to 31 March 2001, a total of 498 patients were screened for inclusion. 419 patients fulfilled all eligibility criteria and were included in the study (Figure 1). Of the 419 included patients, 36 patients were also included in a study of depression [33], and 23 patients were included in a study of low back pain [34]. The last patient follow-up ensued on 12 April 2005. Included and not included patients did not differ significantly regarding age, gender, diagnosis, disease duration, baseline Disease Score, or baseline Symptom Score.

The total number of patients eligible for screening (i.e. patients referred to EYT) during the recruitment period was estimated at approximately 2000 patients. We tested the hypothesis that the extent of patient selection by each physician (= the proportion of eligible vs. included patients) would correlate positively with clinical outcomes. The proportion was median 2.8 (interquartile range (IQR) 0.6–7.9, n = 52 physicians). There was a weak correlation between this proportion and the 0–12 month improvement of Disease Score (Spearman-Rho 0.19, p = 0.014 n = 168 patients) and no significant correlation with the improvement of Symptom Score (Spearman-Rho -0.07, p = 0.311, n = 225 patients).

75.4% (316/419) of patients were enrolled by general practitioners, 10.0% by paediatricians, 4.5% by internists, and 10.0% by other specialists. The physicians' setting was primary care practice (87.8% of patients, n = 368/419), referral practice (8.6%), and outpatient clinic (3.6%).

97.4% (408/419) of patients returned at least one follow-up questionnaire. The 12-month questionnaire was returned by 87.6% of patients; these patients did not differ significantly from non-respondents (12.4%) regarding age, gender, diagnosis, disease duration, baseline Disease Score, and baseline Symptom Score. Corresponding dropout analyses for the 24-month follow-up also showed no differences. The physician follow-up documentation was available for 84.7% (355/419) of patients after six months and for 77.2% after 12 months.

### Baseline characteristics

Most frequent main diagnoses, classified by ICD-10 (International Classification of Diseases, Tenth Edition), were F00-F99 Mental Disorders (31.7%, 133/419 patients), M00-M99 Musculoskeletal Diseases (23.4%), and J00-J99 Respiratory Diseases (7.6%). Most frequent single diagnoses were back pain/sciatica (8.1%, 34/419 patients), neck-shoulder-arm pain (7.6%), depression (6.4%), fatigue (6.2%), childhood emotional disorder (3.8%), headache/migraine (3.3%), and asthma (3.1%).

Median disease duration was 3.0 years (IQR 1.0–8.5); in 97.9% (410/419) of patients disease duration was six weeks or longer. The patients had median 1.0 (IQR 0.0–2.0) comorbid diseases. Most common comorbid diseases, classified by ICD-10, were F00-F99 Mental Disorders (14.1%, 91 out of 645 diagnoses), M00-M99 Musculoskeletal Diseases (12.4%), E00-E90 Endocrine, Nutritional and Metabolic Diseases (9.5%), and I00-I99 Circulatory Diseases (8.5%).

The patients were recruited from 13 of 16 German federal states. Median age was 38.0 years (IQR 14.0–48.0, mean 34.8 years). Compared to the German population, socio-demographic items were more favourable for education, occupation, alcohol, smoking, and overweight; items were similar for unemployment, low-income, living alone, severe disability status, sport, underweight; and were less favourable for work disability pension and sick-leave (Table 1).

**Table 1:** Socio-demographic data

		**Study patients**		**German primary care patients**	
**Items**		**N**	**Percent**	**Percent**	**Source**

Female gender		297/419	71%	53%	[43]
Age groups	0–19 years	111/419	26%	14%	[43]
	20–39 years	115/419	27%	27%	[43]
	40–59 years	150/419	36%	27%	[43]
	60–75 years	43/419	10%	21%	[43]

		**Adult study patients enrolled after 1 Jan 1999**		**German population**	

"Fachhochschule" or university entrance qualification		172/280	61%	19%	[44]
University degree		76/279	27%	6%	[44]
Wage earners		8/280	3%	18%	[44]
Unemployed during last 12 months	Economically active patients	7/147	5%	10%	[44]
Living alone		57/278	20%	21%	[44]
Net family income < 900 € per month		33/231	14%	16%	[44]
Alcohol use daily (EYT) vs. almost daily (Germany)	Male	1/53	2%	28%	[45]
	Female	7/227	3%	11%	
Regular smoking	Male	9/53	17%	37%	[46]
	Female	40/226	18%	28%	
Sports activity ≥ 1 hour weekly	Age 25–69	116/257	45%	39%	[47]
Body mass index < 18.5 (low)	Male	4/53	8%	1%	[48]
	Female	12/223	5%	4%	
Body mass index ≥ 25 (overweight)	Male	8/53	15%	56%	[48]
	Female	69/223	31%	39%	
Permanent work disability pension		20/279	7%	3%	[49]
Severe disability status		24/279	9%	12%	[50]
Sick leave days in the last 12 months, mean (SD)	Economically active patients	33.0 (68.3) days		17.0 days	[51]

### Therapies

EYT administration was documented during the first 24 months after study enrolment. In this period, 93.6% (392/419) of patients had EYT; 2.9% did not have EYT; for 3.6% EYT documentation is incomplete or inconclusive. EYT started median 15 (IQR 4–41) days after enrolment. Median therapy duration was 119 (IQR 84–188) days, median number of therapy sessions was 12 (IQR 10–19). At the last documented EYT session, further EYT sessions were scheduled for 14% (49/344) of evaluable patients. During the first six months after study enrolment 72.1% (302/419) of patients used AM medication and 1.4% (6/419) had AM art therapy.

Non-AM adjunctive therapies, health services, and sick leave are listed in Table 2, together with AM medication. Comparing the pre-study year to the first and second study year, respectively, the only consistent change over both years was an increase in psychotherapy by average one session per patient. In the first study year AM medication use and the number of physician and dentist visits increased, and in the second year the number of rehabilitation days and non-AM medication use decreased, compared to the pre-study year. The remaining items did not change significantly.

**Table 2:** AM medication, non-AM adjunctive therapies, health service use, and sick leave days

**Item**	**Pre-study year**	**0–12 months**			**12–24 months**		
	**Mean (SD)**	**Mean (SD)**	**Median difference (95%-CI) from pre-study year**	**P value**	**Mean (SD)**	**Median difference (95%-CI) from pre-study year**	**P value**

AM medicines per day	0.45 (0.80)	0.70 (0.90)	0.24 (0.17 to 0.37)	p < 0.001	0.40 (0.71)	-0.02 (-0.08 to 0.04)	p = 0.505
Non-AM medicines/day	0.65 (0.90)	0.69 (0.94)	0.01 (-0.04 to 0.06)	p = 0.628	0.59 (0.88)	-0.06 (-0.13 to -0.01)	p = 0.032
Physician and dentist visits	18.12 (21.19)	18.82 (16.03)	1.24 (0.19 to 2.50)	p = 0.028	18.67 (50.41)	-1.43 (-2.50 to 0.00)	p = 0.041
Paraclinical investigations	5.70 (6.66)	5.75 (6.77)	0.00 (-0.62 to 0.50)	p = 0.737	5.24 (6.71)	-0.50 (-1.00 to 0.00)	p = 0.093
Hospital days	3.42 (14.72)	2.57 (10.91)	-1.10 (-5.00 to 1.46)	p = 0.346	2.04 (7.42)	-0.04 (-2.32 to 1.18)	p = 0.929
Rehabilitation days	2.02 (8.36)	1.76 (7.48)	0.00 (-10.02 to 7.46)	p = 0.921	1.55 (6.20)	-0.69 (-0.97 to -0.62)	p = 0.005
Surgeries	0.19 (0.51)	0.14 (0.41)	0.00 (-0.47 to 0.00)	p = 0.323	0.12 (0.38)	0.00 (-0.42 to 0.07)	p = 0.909
Physiotherapy and ergotherapy sessions	8.92 (17.83)	9.25 (22.80)	1.00 (-2.00 to 4.00)	p = 0.425	10.91 (28.35)	-1.22 (-4.19 to 1.31)	p = 0.379
Psychotherapy sessions	2.64 (12.96)	3.54 (9.42)	3.98 (1.50 to 7.00)	p = 0.008	3.56 (10.34)	2.68 (1.67 to 3.67)	p < 0.001
Sick leave days*	32.97 (68.26)	34.61 (80.65)	3.50 (-2.00 to 8.00)	p = 0.185	29.85 (68.69)	3.18 (-2.18 to 8.00)	p = 0.210
Patients with Heilpraktiker visit (n + %)**	32/250 (12.8%)	29/250 (11.6%)		p = 0.710	27/250 (10.8%)		p = 0.511

Patients enrolled after 1 Jan 1999 with at least 3 of 5 follow-ups (n = 339). *Patients engaged in economic activity (n = 128). **Patients with complete data for all time periods.

Use of diagnosis-related adjunctive therapies (see Methods) within the first six study months was analysed in patients with a main diagnosis of mental, respiratory or musculoskeletal diseases, or headache disorders (n = 278). Out of 251 evaluable patients, 63% (n = 157) had no diagnosis-related adjunctive therapy.

### Clinical outcomes

**Figure 2:**
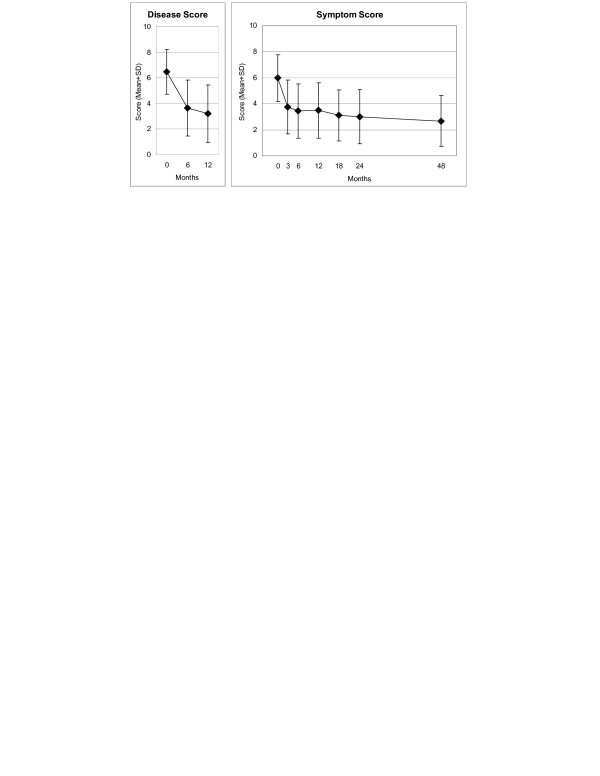
**Disease and Symptom Scores**. Disease Score: physicians' assessment, Symptom Score: patients' assessment. Range 0 "not present", 10 "worst possible".

**Figure 3:**
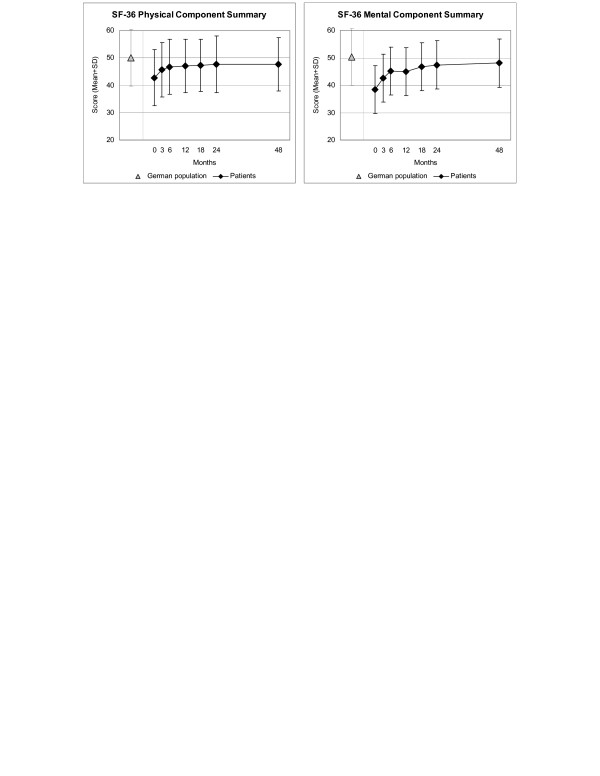
**SF-36 Physical and Mental Component Summary Measures**. Higher scores indicate better health. Adult patients and German population (standardised for age and gender) [28]

**Figure 4:**
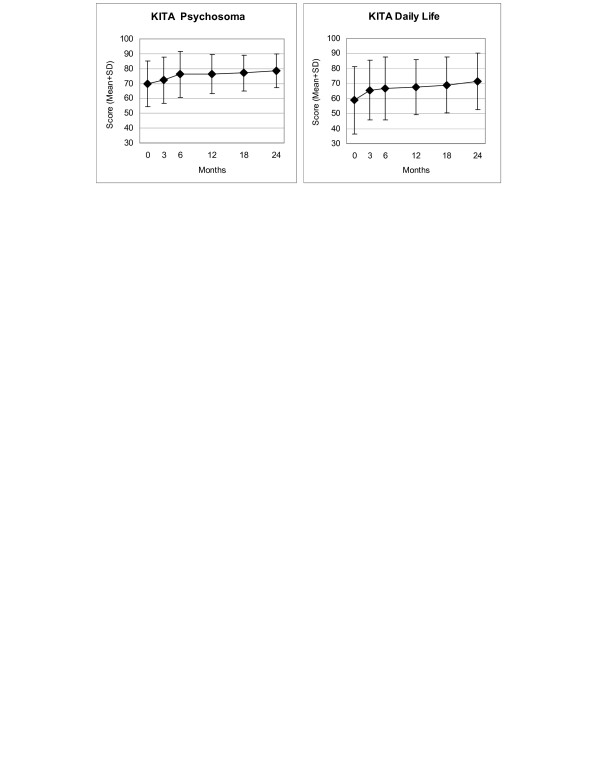
**KITA Psychosoma and Daily Life subscales**. Range 0–100, higher scores indicate better health. Children aged 1–7 years.

Disease and Symptom Scores (Figure 2), all eleven SF-36 scores (adults, Figure 3), and both KITA subscales (children aged 1–7, Figure 4) improved significantly between baseline and all subsequent follow-ups (except KITA Psychosoma in the first three months). For all these 15 outcomes, the most pronounced improvement occurred during the first six months. After 12 months, Disease and Symptom Scores were improved from baseline in 86.9% and 83.6% of patients, respectively (Table 3); an improvement of ≥50% of baseline scores was observed in 61.2% (145/237 evaluable patients) and 46.4% (156/336), respectively. Disease and Symptom Scores improved similarly in male and female adults, in children, and in the seven most common diagnosis groups. Effect sizes for the 0–12 month comparison were large for Disease and Symptom Scores (1.34 and 1.04) and small-to-medium (range 0.41–0.67) for the SF-36 and KITA scores (Table 3). All these improvements were maintained until the last follow-up.

**Table 3:** Clinical outcomes 0–12 months

**Item**	**N**	**0 months**	**12 months**	**0 months vs. 12 months**			
		**Mean (SD)**	**Mean (SD)**	**P-value**	Median difference (95%-CI)*	**Improved**	**SRM**

Disease Score (0–10)	237	6.65 (1.81)	3.19 (2.27)	p < 0.001	4.00 (3.50 to 4.00)	87%	1.34
Symptom Score (0–10)	336	5.95 (1.75)	3.49 (2.12)	p < 0.001	2.50 (2.25 to 2.75)	84%	1.04
SF-36 scales (0–100)							
-Physical Function	270	75.34 (22.74)	83.18 (19.41)	p < 0.001	10.00 (7.50 to 10.00)	63%	0.42
-Role Physical	267	42.51 (39.20)	67.79 (37.20)	p < 0.001	37.50 (37.50 to 50.00)	55%	0.63
-Role-Emotional	268	47.26 (41.87)	70.58 (38.09)	p < 0.001	33.34 (33.30 to 50.00)	49%	0.55
-Social Functioning	272	62.13 (25.75)	75.28 (24.37)	p < 0.001	18.75 (12.50 to 25.00)	58%	0.49
-Mental Health	271	54.21 (18.65)	65.05 (19.00)	p < 0.001	12.00 (8.00 to 14.00)	71%	0.57
-Bodily Pain	272	55.91 (28.41)	66.93 (27.65)	p < 0.001	16.00 (11.50 to 20.00)	55%	0.41
-Vitality	271	38.68 (17.85)	51.49 (18.68)	p < 0.001	15.00 (12.50 to 17.50)	68%	0.67
-General Health	268	50.86 (18.80)	58.39 (19.55)	p < 0.001	8.50 (6.00 to 10.00)	65%	0.44
SF-36 Health Change (1–5**)	272	3.23 (1.08)	2.15 (1.09)	p < 0.001	1.50 (1.00 to 1.50)	69%	0.68
SF-36 Physical Component	263	43.13 (10.25)	47.10 (9.78)	p < 0.001	3.90 (2.83 to 4.97)	68%	0.44
SF-36 Mental Component	263	38.31 (11.67)	45.01 (11.76)	p < 0.001	6.45 (4.94 to 7.96)	69%	0.55
KINDL subscales (0–100)							
-Psychic	35	67,36 (15,27)	70,68 (15,64)	p = 0.188	3.41 (-2.27 to 9.09)	60%	0.20
-Somatic	35	70,57 (14,47)	75,60 (9,35)	p = 0.071	4.17 (0.00 to 9.72)	66%	0.37
-Social	35	69,90 (11,95)	73,16 (11,78)	p = 0.063	4.17 (0.00 to 7.29)	66%	0.28
-Function	33	64,39 (14,33)	67,94 (10,44)	p = 0.187	3.41 (-2.27 to 7.96)	61%	0.25
KINDL Summary Score (0–100)	35	67.86 (11.02)	71.48 (9.79)	p = 0.063	3.59 (-0.07 to 7.65)	63%	0.34
KITA subscales (0–100)							
-Psychosoma	51	69.53 (15.45)	77.21 (13.60)	p = 0.001	9.38 (4.17 to 12.50)	69%	0.51
-Daily Life	56	59.23 (21.78)	68.14 (18.52)	p = 0.001	10.42 (4.17 to 14.58)	63%	0.53

*Positive differences indicate improvement. Improved: Percentage of patients improved from baseline. **1 = "much better now than one year ago", 5 = "much worse now than one year ago". SRM: Standardised Response Mean effect size (small: 0.20–0.49, medium: 0.50–0.79, large: ≥ 0.80).

**Figure 5:**
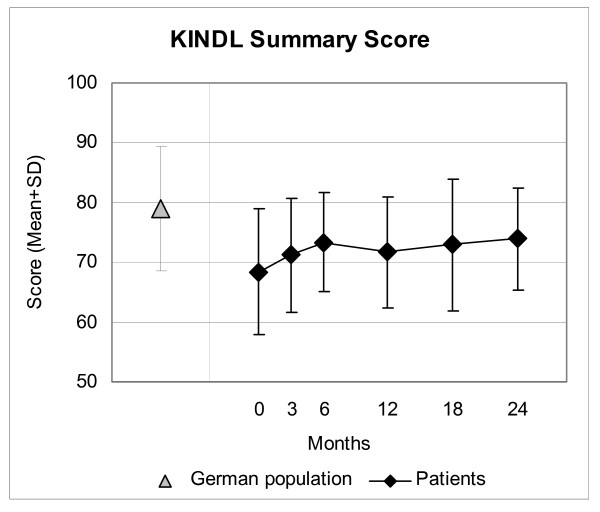
**KINDL Summary Score**. Range 0–100, higher scores indicate better health. Children aged 8–16 years and German population sample (9–12 years) [29].

In children aged 8–16, KINDL Summary Score (Figure 5) as well as KINDL Psychic and Somatic subscales improved significantly between baseline and the six-month, 18-month (except KINDL Somatic subscale), and 24-month follow-ups, respectively. KINDL Social and Function subscales did not change significantly during the study.

We performed two post-hoc sensitivity analyses of 0–12 month Disease and Symptom Score outcomes. The first analysis concerned dropout bias. The main analysis had comprised all patients with evaluable data at baseline and 12-month follow-up. In the first sensitivity analysis, missing values after 12 months were replaced with the last value carried forward, reducing the average 0–12 month improvements by 19% (3.46→2.78 points) and 4% (2.46→2.35 points), respectively. The second analysis concerned the effects of relevant adjunctive therapies, and was performed on patients with a main diagnosis of mental, respiratory or musculoskeletal diseases or headache disorders. Restricting this sample to patients not using diagnosis-related adjunctive therapies during the first six study months (see Methods), the average Disease and Symptom Score improvements were increased by 10% (3.55→3.96 points) and 6% (2.23→2.36 points), respectively.

### Other outcomes

At six-month follow-up, patients' average therapy outcome rating (numeric scale from 0 "no help at all" to 10 "helped very well") was 7.42 (SD 2.29); patient satisfaction with therapy (from 0 "very dissatisfied" to 10 "very satisfied") was 8.08 (SD 2.19). Patients' EYT effectiveness rating was positive ("very effective" or "effective") in 86.1% (315/366) of patients, and negative ("less effective", "ineffective" or "not evaluable") in 13.9%. Physicians' effectiveness rating was positive in 79.3% (264/333) and negative in 20.7%. Ratings of therapy outcome, satisfaction, and effectiveness did not differ significantly between adults and children, or between six- and 12-month follow-ups.

During the first 24 study months adverse reactions to EYT occurred in 3.1% (13/419) of patients. Three (0.7%) patients had adverse reactions of severe intensity (symptom aggravation, inner tension, depressed mood), no patient stopped EYT due to adverse reactions. One child had adverse reactions (moderate restlessness) to adjunctive AM massage therapy, which was stopped. Four patients had adverse reactions to non-AM therapies. Adverse reactions from AM medications occurred in 5.3%, (18/337) of users, adverse reactions from non-AM medication occurred in 12.8% (46/358) of users (p < 0.001).

Nine patients had Serious Adverse Events. Three patients were acutely hospitalised and six patients died: five from malignant disease and one patient, hospitalised for severe depression, from an accident, possibly suicide. None of these Serious Adverse Events were related to any therapy or medication.

## Discussion

This is the first large study focusing on EYT. We aimed to obtain information on EYT under routine conditions in Germany and studied clinical outcomes in outpatients referred to EYT for chronic diseases. The study was conducted in conjunction with a health insurance program providing EYT regardless of diagnosis. For this reason, and because the range and frequency of indications for EYT in outpatient care was largely unknown prior to the study, we included patients of all ages with all diagnoses. Most frequent indications were mental and musculoskeletal disorders. Following EYT (and adjunctive AM medication), significant improvements of disease symptoms and quality of life were observed. The largest improvements (large effect sizes, half of patients improved by at least 50% of their baseline scores) were observed for the items which directly measure the conditions treated with EYT, i.e. Disease and Symptom Scores. The improvements were maintained during the four-year follow-up and were not accompanied by an increase of adjunctive therapies, except for a small increase in psychotherapy use.

### Strengths and limitations

Strengths of this study include a large patient sample, a long follow-up period, high follow-up rates, and the participation of 30% of all AM-certified physicians and EYT therapists in Germany. The participating physicians and therapists resembled all eligible physicians/therapists with respect to socio-demographic characteristics, and included patients resembled not included, screened patients regarding baseline characteristics. These features suggest that the study to a high degree mirrors contemporary EYT practice. Moreover, in the present early phase of EYT evaluation, the inclusion of all diagnoses is an advantage, offering a comprehensive picture of EYT practice. On the other hand, it was not feasible to have disease-specific outcomes for all diagnoses included. Nonetheless, the larger AMOS project, of which this study is part, included disease-specific outcomes for major disease groups [33,34].

Since the study had a long recruitment period, the participating physicians were not able to screen and include all their eligible patients (patients referred to EYT). It was estimated that physicians enrolled every third patient referred to EYT. This selection could bias results if physicians were able to predict therapy response and if they preferentially screened and enrolled such patients for whom they expected a particularly favourable outcome. In this case one would expect the degree of selection (= the proportion of referred vs. enrolled patients) to correlate positively with clinical outcomes. There was, however, only a weak correlation with Disease Score (+0.19) and a no significant correlation with Symptom Score. These analyses do not suggest that physicians' screening of patients referred to EYT was affected by selection bias.

A limitation of the study is the absence of a comparison group receiving another treatment or no therapy. Accordingly, for the observed improvements one has to consider several other causes apart from EYT: Non-AM adjunctive therapies cannot explain the improvements of Disease and Symptom Scores, since the improvements were even more pronounced in patients not using such therapies (analysed in patients with mental, respiratory or musculoskeletal disease or headache syndromes, together comprising 66% of the study sample). Dropout bias could explain up to 19% of the 0-12-month improvement of Disease Score but only 4% of the corresponding Symptom Score improvement. Natural recovery and regression to the mean, which could also bias results, will be addressed in a separate analysis (Hamre et al, submitted for publication). Other possible confounders are AM medication (which was used by three-fourth of patients), observation bias, and psychological factors like patient expectations. Since, however, EYT was evaluated as a therapy package, the question of specific therapy effects vs. non-specific effects (placebo effects, context effects, patient expectations etc.) was not an issue of the present analysis.

Since EYT was to be evaluated under routine conditions, therapy was administered at the discretion of the physicians and EYT therapists, and not according to a standardised protocol. This raises the question of whether study interventions would be replicable in future studies. However, EYT therapists worldwide are trained according to a highly standardised curriculum, specifying individual EYT movements for specific diseases, constitution types, and movement patterns. Therefore, relevant therapy differences across settings would not be expected. Moreover, in this study, any local therapy differences would probably be offset by the large number of participating EYT therapists. Nevertheless, a limitation of our study is that the specific EYT movements selected for each patient were not documented.

### Study implications

This study confirms previous studies of the characteristics of AM users [15,35-38]: Patients are predominantly middle-aged women or children, education and occupation levels are higher than average, and typical indications are mental and musculoskeletal disorders. Previous studies conducted in inpatient [16-24] and outpatient clinics [24,25] have evaluated AM therapy including EYT for rheumatoid arthritis [16], asthma [24], hepatitis C [17,25], breast cancer [18], anorexia nervosa [19], lumbar disc disease [20], chronic musculoskeletal pain [21], and in the rehabilitation after stroke [22] and myocardial infarction [23]. All these studies had some favourable outcomes; the three largest studies (range 60–81 AM patients) found improved quality of life in breast cancer patients [18]; high anorexia nervosa cure rates [19]; and reduced pain, reduced use of non-steroidal anti-inflammatory drugs and muscle relaxants, and earlier return to work in lumbar disc disease [20].

In accordance with these findings from secondary care, our predominantly primary care study of EYT users demonstrated long-standing improvements in disease symptoms and quality of life across a range of conditions. Most common indications for EYT were musculoskeletal pain, depression, fatigue, childhood emotional disorder, and headache disorders. For these conditions some patients will not profit from standard therapies (drugs, physiotherapy, psychotherapy, multimodal inpatient therapies, surgery), e.g. between three and five patients must be treated with drugs for one patient to benefit [39-42]. Other patients discontinue standard therapies due to adverse reactions or reject them because therapies are passive (e.g. drugs, passive physiotherapy) or can be felt as intrusive, too verbal (psychotherapy) or too mechanical-repetitive (exercise physiotherapy). Thus, for patients where standard therapies are not preferred or tolerated well, or do not cure, EYT as a non-verbal artistic exercising therapy is a promising treatment option.

## Conclusion

In this study, patients practising EYT exercises had long-term reduction of chronic disease symptoms and improvement of quality of life, without relevant increase in health service use. Although the pre-post design of the present study does not allow for conclusions about comparative effectiveness, study findings suggest that EYT can be useful for patients motivated for this therapy.

## Abbreviations

AM: anthroposophic medicine, AMOS: Anthroposophic Medicine Outcomes Study, EYT: eurythmy therapy, IQR: interquartile range.

## Competing interests

Within the last five years HJH has received restricted research grants from the pharmaceutical companies Weleda and Wala, who produce AM medications. Otherwise all authors declare that they have no competing interests.

## Authors' contributions

HJH, CMW, SNW, and HK contributed to study design. HJH, AG, and HK contributed to data collection. HJH, RZ, and HK wrote the analysis plan, HJH and AG analysed data. HJH was principal author of the paper, had full access to all data, and is guarantor. All authors contributed to manuscript drafting and revision and approved the final manuscript.

## Pre-publication history

The pre-publication history for this paper can be accessed here:


